# The *GSTP1 105Val* Allele Increases Breast Cancer Risk and Aggressiveness but Enhances Response to Cyclophosphamide Chemotherapy in North China

**DOI:** 10.1371/journal.pone.0067589

**Published:** 2013-06-24

**Authors:** Jie Ge, Ai-Xian Tian, Qing-Shan Wang, Peng-Zhou Kong, Yue Yu, Xiao-Qing Li, Xu-Chen Cao, Yu-Mei Feng

**Affiliations:** 1 Department of Biochemistry and Molecular Biology, Tianjin Medical University Cancer Institute and Hospital, Tianjin, China; 2 Department of Breast surgery, Tianjin Medical University Cancer Institute and Hospital, Tianjin, China; 3 Key Laboratory of Breast Cancer Prevention and Treatment of the Ministry of Education, Tianjin Medical University Cancer Institute and Hospital, Tianjin, China; The Ohio State University, United States of America

## Abstract

The glutathione-S-transferase (GST) family contributes to the inactivation of various toxic compounds formed as secondary metabolites during oxidative stress. GSTP1 accounts for the majority of the GST family enzymatic activity, and the activity of GSTP1 enzyme can be altered by the presence of the *Ile105Val* polymorphism. In this study, we examined the polymorphic frequency of *GSTP1 Ile105Val* genotype in 920 breast cancer patients and 783 healthy controls in women of North China. Results showed that *GSTP1 105Val* allele (*Ile*/*Val* and *Val*/*Val*) was associated with a higher breast cancer risk (OR = 1.38, 95% CI: 1.14–1.69; *P* = 0.001) and more aggressive tumors with histological grade III (OR = 1.15, 95% CI: 1.05–1.26; *P* = 0.001), lymph node metastases (OR = 2.35, 95% CI: 1.72–3.21; *P* < 0.001), as well as ER negative (OR = 1.77, 95% CI: 1.31–2.39; *P* < 0.001) than those carrying the *Ile*/*Ile* allele. However, the patients with the *GSTP1 105Val* genotype had a better disease free survival after cyclophosphamide (CTX)-based chemotherapy than those with *Ile*/*Ile* (HR = 0.77, 95% CI: 0.45–0.91; *P* < 0.001). Furthermore, *in vitro* cellular experiments demonstrated that breast cancer cells with the *GSTP1 105Val* allele were significantly more sensitive to CTX-induced proliferation inhibition. Thus, we conclude that the *GSTP1 105Val* allele increases breast cancer risk and aggressiveness and enhance response to CTX-based chemotherapy in women of North China. Detection of the *GSTP1 Ile105Val* genotype may help screen for high-risk populations and direct individualized therapy.

## Introduction

Breast cancer is the most common malignancy and the second leading cause of cancer-related death in women worldwide [1]. In China, an increasingly modern lifestyle has been accompanied by a sharp jump in the breast cancer rate in urban areas. Clinical evidence has shown that women at the same pathologic stages of cancer undergoing the same treatment may have different outcomes [2]. In addition to the known risk factors of age, family history, age at childbirth, menopause and hormone therapy, the individual genetic variability also impacts drug metabolism and subsequent efficacy [3]. Chemotherapy has been established as the standard of care for breast cancer patients [4,5], especially those with locally advanced breast cancer. Among the chemotherapy regimens, CTX-based chemotherapy is recommended by the National Comprehensive Cancer Network (NCCN) as the clinical practice guidelines for breast cancer. Although chemotherapy improves disease-free survival (DFS) and overall survival (OS) of breast cancer patients [6], it is challenging to identify the patients who will benefit from chemotherapy and reduce the use of chemotherapy in those who will not benefit.

Glutathione-S-transferases (GSTs), a superfamily of dimeric phase II metabolic enzymes, are divided into six classes and play important roles in the metabolism of products of oxidative stress including by-products of lipid and DNA oxidation [7–9]. *GSTP1* encodes the π-class of enzymes which accounts for approximately 90% of the enzymatic activity of the GST family, and its expression is found in many normal and malignant tissues [10]. The GSTP1 enzymatic activity can be altered by genetic polymorphisms. The *GSTP1 Ile105Val* (rs1695 or rs947894) single-nucleotide polymorphism (SNP) is a transition from an A to a G at nucleotide position 313 (A313G), leading to an Ile105Val amino acid change located near the substrate binding site of the enzyme [11]. The altered protein expression may lead to subsequent development of a malignant phenotype, whereas may enhance chemotherapy efficacy [11–13]. To date, researches have reported the correlation of *GSTP1 Ile105Val* polymorphisms with breast cancer risk and chemosensitivity [14,15] Several studies have reported that women with the *GSTP1 105Val* genotype in Shanghai of Southeast China [15,16], America [17], and India [18,19] have greater breast cancer risks. However, some studies have reported conflicting results in women from Italy [20] and Australia [21]. Thus, the role of *GSTP1* in breast cancer is also controversial. In particular, the association of *GSTP1 Ile105Val* genetic polymorphisms with tumor aggressiveness and response to cyclophosphamide (CTX) and CTX-based chemotherapy remains unidentified.

Among individuals with similar GSTP1 expression levels in somatic cells, enzyme catalytic activity would be expected to vary according to the presence of variant *GSTP1* genotypes. We speculate that the breast cancer tumors with the *GSTP1 105Val* variant genotype may have different biological characteristics and responses to CTX-based treatment because of altered enzymatic activity, which may ultimately lead to survival differences of patients. In current study, we examined the distribution frequencies of the *GSTP1 Ile105Val* genotype in breast cancer patients and age-matched healthy women of North China and evaluated the association of the genotypes with breast cancer risk, tumor aggressiveness and the survival of patients treated with a CTX-based regimen. Furthermore, we validated the differences of individuals with the *GSTP1 105Ile* and *Val* alleles in response to CTX cellular cytotoxicity through *in vitro* experiments.

## Results

### Genotypic distribution of *GSTP1 Ile105Val* in breast cancer patients and control subjects

The alleles and genotypic frequencies of *GSTP1 Ile105Val* in the control population and patients are shown in Table 1. The genotypic frequencies of *GSTP1 Ile105Val* in the patient population were 58.7% of *Ile*/Ile, 35.3% of *Ile*/*Val* and 6.0% of *Val*/*Val*, while in the control group, the genotypic frequencies of *GSTP1 Ile105Val* genotypes were 66.3% of *Ile*/*Ile*, 29.3% of *Ile*/*Val* and 4.4% of *Val*/*Val*. There were no deviations from Hardy-Weinberg Equilibrium in either the cases or controls (*P* > 0.05).

**Table 1:** The genotypic frequencies of the *GSTP1 Ile105Val* SNP in women of North China: the breast cancer versus control groups.

Genotype of *GSTP1* ** *Ile105Val*	Cases (%) n = 920	Controls (%) n = 783	OR (95% CI)	*P*-value
*Ile*/*Ile*	540 (58.7)	519 (66.3)	1.00	
*Ile*/*Val*	325 (35.3)	230 (29.3)	1.36 (1.10-1.67)	0.004
*Val*/*Val*	55 (6.0)	34 (4.4)	1.55 (1.00-2.42)	0.050
*Ile*/*Val* and *Val*/*Val*	380 (41.3)	264 (33.7)	1.38 (1.14-1.69)	0.001

### Association between *GSTP1 Ile105Val* genotype and breast cancer risk

The comparison between control and breast cancer patient subjects revealed a significant difference among the three *GSTP1 Ile105Val* genotypes (*Ile*/*Ile*, *Ile*/*Val* and *Val*/*Val*). Logistic regression analysis showed that women carrying the *Ile*/*Val* and *Val*/*Val* had a OR of 1.36 (95% CI: 1.10-1.67; *P* = 0.004) and 1.55 (95% CI: 1.00-2.42; *P* = 0.050), respectively, suggesting that genotypes with the *Val* allele (*Ile/Val* and *Val/Val*) led to an increased risk of breast cancer development (OR = 1.38, 95% CI: 1.14-1.69; *P* = 0.001; Table 1).

### Association between *GSTP1 Ile105Val* genotypes and clinicopathological characteristics

To further characterize the significance of the *GSTP1 Ile105Val* genotypes in breast cancer, the associations with various clinicopathological characteristics including patient age, clinical staging, histopathological grading, as well as ER, PR and HER2 status, were analyzed. The results showed that the *Ile/Val* and *Val/Val* genotypes of *GSTP1 Ile105Val* significantly correlated with patient age, histological grade, lymph node involvement, and ER status. The tumors with the *Val* allele more frequently were histological grade III (OR = 1.15, 95% CI: 1.05-1.26; *P* = 0.001), ER negative (OR = 1.77, 95% CI: 1.31-2.39; *P* < 0.001), as well as involved lymph node metastases (OR = 2.35, 95% CI: 1.72-3.21; *P* <0.001) than tumors with the *Ile*/*Ile* allele. No further significant associations were observed between the SNP genotypes and other clinic pathological features (Table 2).

**Table 2:** The association between *GSTP1 Ile105Val* genotype and clinicopathological characteristics.

Characteristics	Genotype of *GSTP1* ** *Ile105Val*			
	*Ile*/*Ile* (%)	*Ile*/*Val* (%)	*Val*/*Val* (%)	*Ile*/*Val* and *Val*/*Val* (%)
**Age (year)**				
>=55/<55	180 (54.1)/360 (61.3)	129 (38.7)/196 (33.4)	24 (7.2)/31 (5.3)	153 (45.9)/227 (38.7)
OR (95% CI)	1.00	1.32 (0.99-1.75)	1.55 (0.88-2.72)	1.35 (1.03-1.77)
*P*-value		0.059	0.125	0.031
**Histological grade**				
III/I+II	112 (51.6)/388 (64.1)	103 (47.5)/202 (33.4)	32 (14.7)/15 (2.5)	105 (48.4)/217 (35.9)
OR (95% CI)	1.00	1.17 (1.07-1.29)	2.43 (1.60-3.70)	1.15 (1.05-1.26)
*P*-value		0.001	< 0.001	0.001
**Clinical stage**				
III+IV/I+II	92 (55.4)/447 (62.3)	62 (37.3)/230 (32.1)	12 (7.2)/40 (5.6)	74 (44.6)/270 (37.6)
OR (95% CI)	1.00	1.31 (0.92-1.88)	1.46 (0.74-2.89)	1.33 (0.95-1.87)
*P*-value		0.140	0.277	0.099
**Lymph node involvement**				
Pos. / Neg.	98 (44.7)/424 (65.5)	99 (45.2)/193 (29.8)	22 (10.0)/30 (4.6)	121 (55.3)/223 (34.5)
OR (95% CI)	1.00	2.22 (1.60-3.08)	3.17 (1.76-5.74)	2.35 (1.72-3.21)
*P*-value		0.001	< 0.001	< 0.001
**ER status**				
Neg. / Pos.	114 (46.0)/340 (60.1)	104 (41.9)/206 (36.4)	30 (12.1)/20 (3.5)	134 (54.0)/226 (39.2)
OR (95% CI)	1.00	1.51 (1.10-2.07)	4.47 (2.45-8.19)	1.77 (1.31-2.39)
*P*-value		0.011	< 0.001	< 0.001
**PR status**				
Neg. / Pos.	132 (57.1)/334 (57.3)	83 (35.9)/225 (38.6)	16 (6.9)/24 (4.1)	99 (42.8)/249 (42.7)
OR (95% CI)	1.00	0.93 (0.68-1.29)	1.69 (0.87-3.28)	1.01 (0.74-1.37)
*P*-value		0.675	0.119	0.970
**HER2 status**				
Pos. / Neg.	106 (56.4)/338 (54.0)	68 (36.2)/252 (40.3)	14 (7.4)/36 (5.8)	82 (43.6)/288 (46.0)
OR (95% CI)	1.00	0.86 (0.61-1.22)	1.24 (0.64-2.39)	0.91 (0.65-1.26)
*P*-value		0.394	0.519	0.564

### Association between the *GSTP1 Ile105Val* genotype and DFS in breast cancer patients

**Figure 1:**
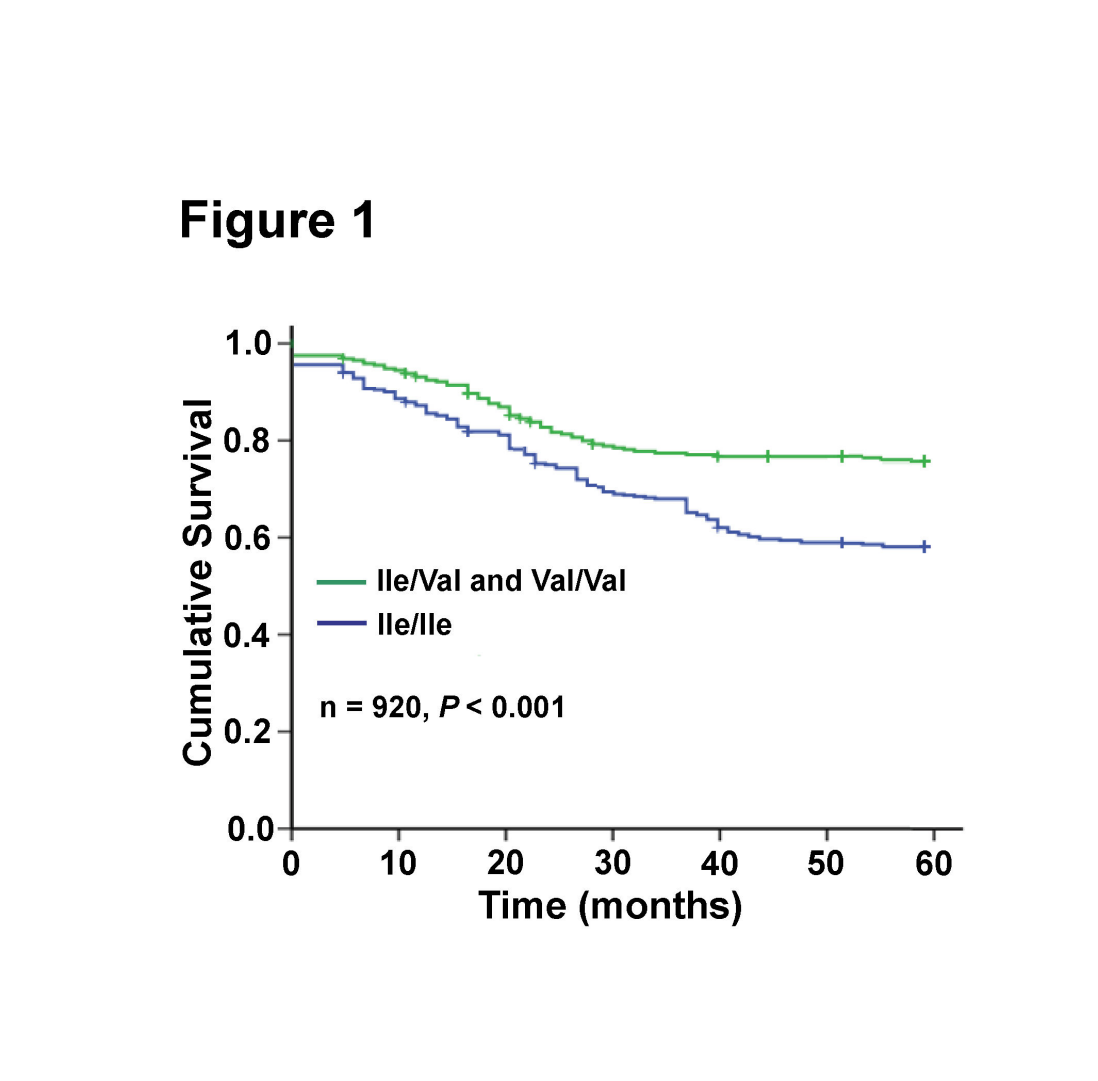
Kaplan-Meier analysis of DFS among patients with *GSTP1* *Ile105Val* genotypes treated with a CTX-based regimen.

To analyze the relationship between the *GSTP1 Ile105Val* genotype of breast cancer patients and their prognosis after CTX-based chemotherapy, we compared the 5-year DFS rate between patients with *Val* allele and those with *Ile*/*Ile* genotype. The results indicated that the 5-year DFS rate of patients with the *Val* allele was higher than those with the *Ile*/*Ile* genotype (HR = 0.76, 95% CI: 0.61-0.93; *P* = 0.008; Table 3). Further Kaplan-Meier DFS analysis showed that the *GSTP1* genotype was associated with DFS after analysis with the Cox proportional hazards model (Figure 1).

**Table 3:** The association between the *GSTP1 Ile105Val* genotype and 5-year DFS (n = 879).

Genotype of *GSTP1* ** *Ile105Val*	Cases	5-year DFS	HR (95% CI)	*P*-value
*Ile/Ile*	518	322	1.00	
*Ile/Val*	309	251	0.77 (0.62-0.95)	0.017
*Val/Val*	52	46	0.70 (0.46-1.07)	0.102
*Ile/Val and Val/Val*	361	297	0.76 (0.61-0.93)	0.008

### Multivariate analysis for *GSTP1 Ile105Val* genotype and DFS in breast cancer patients

To determine whether the *GSTP1 Ile105Val* genotype is an independent factor associated with DFS in breast cancer patients, we performed multivariate analyses. Patient characteristics including the *GSTP1 Ile105Val* genotype, age, histological grade, clinical stage, lymph node involvement, ER status, PR status, and HER2 status were first evaluated with a univariate analysis. Only the variables with a *P* < 0.05 in the univariate analysis were included in the multivariate analysis using a backward stepwise Cox proportional hazards regression model (n = 788). Result showed that the *GSTP1 Ile105Val* genotype was an independent factor associated with the DFS of breast cancer patients (RR = 0.77, 95% CI: 0.45-0.91; *P* < 0.001; Table 4).

**Table 4:** Univariate and multivariate analysis of clinicopathological variables affecting 5-year DFS.

Variable	Comparison	RR (95% CI)	*P*-value
**Univariate**			
Age (year)	<55 vs. >=55	1.17 (0.49-2.73)	0.723
Histological grade	III vs. I+II	1.43 (0.26-4.52)	0.842
Clinical stage	III+IV *vs*. I+II	1.77 (1.29-2.43)	< 0.001
Lymph node status	Pos. vs. Neg.	2.41 (1.32-3.48)	< 0.001
ER status	Neg. vs. Pos.	1.32 (0.64-1.62)	0.419
PR status	Neg. vs. Pos.	1.27 (0.80-4.62)	0.217
HER2 status	Pos. vs. Neg.	1.24 (0.82-3.47)	0.180
*GSTP1 Ile105Val*	Ile/Val+Val/Val *vs*. *Ile*/*Ile*	0.46 (0.34-0.61)	< 0.001
**Multivariate** (n = 788)			
Clinical stage	III+IV *vs*. I+II	1.23 (1.19-1.43)	0.002
Lymph node status	Pos. vs. Neg.	1.51 (1.10-3.62)	0.005
*GSTP1 Ile105Val*	Ile/Val+Val/Val *vs*. *Ile*/*Ile*	0.77 (0.45-0.91)	<0.001

### The *GSTP1 Ile105Val* polymorphism affects the breast cancer cell response to CTX

**Figure 2:**
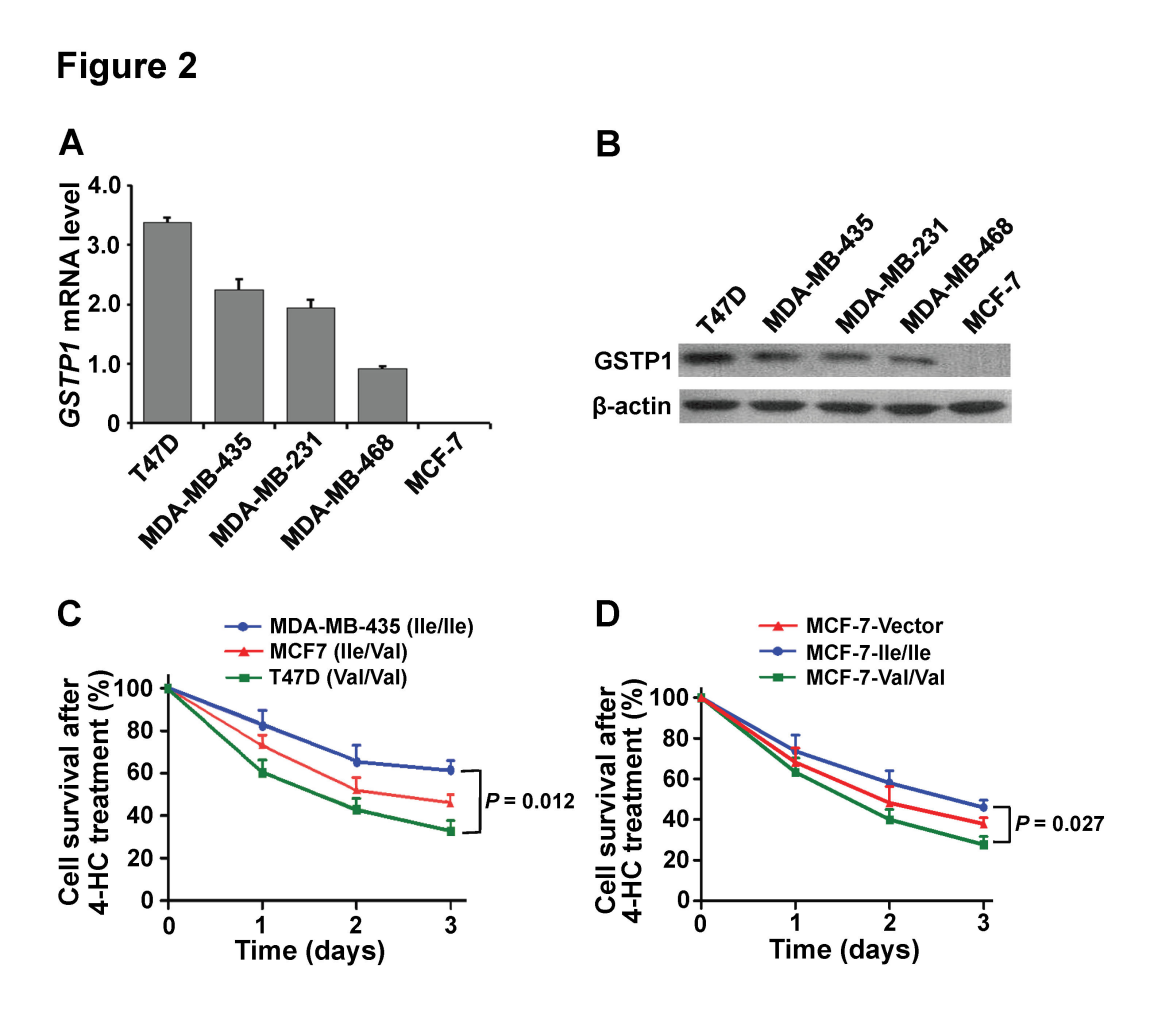
The *GSTP1 Ile105Val* polymorphism affects breast cancer cell response to cyclophosphamide. (**A**) The *GSTP1* mRNA expression levels in breast cancer cell lines were detected using RT-QPCR. (**B**) The GSTP1 protein expression levels in breast cancer cell lines were detected using western blot. (**C**) MDA-MB-435 (Ile/Ile), MCF-7 (Ile/Val), and T47D (Val/Val) cells lines were treated with 4-HC (15 µg/mL) for indicated time, and cell survival rates were analyzed using MTT assay at the indicated time points. (**D**) MCF-7 cells transfected with pEGFP (Vector), pEGFP-GSTP1 105Ile (Ile/Ile), pEGFP-GSTP1 105Val (Val/Val) were treated with 4-HC (15 µg/mL) for indicated time, and cell survival rates were analyzed using MTT assay at the indicated time points. The cells were tested in three independent assays with each containing triplicates and the calculated data of experiments are expressed as mean ± SD.

To investigate the effects of the *GSTP1 Ile105Val* genotype on breast cancer drug resistance *in vitro*, we analyzed the mRNA expression levels, protein levels and the *GSTP1 Ile105Val* genotype in different breast cancer cell lines. The results indicated that *GSTP1* mRNA levels were high in T47D, MDA-MB-435, MDA-MB-231 and MDA-MB-468, but were barely detectable in MCF-7 cells (Figure 2A). The same tendency was found in their protein levels (Figure 2B). The *GSTP1 Ile105Val* genotype was *Val/Val* in T47D, *Ile*/*Ile* in MDA-MB-435, MDA-MB-231 and MDA-MB-468, *Ile/Val* in MCF-7 cells. Since CTX was activated by hepatic cytochrome P450 enzymes and 4-hydroperoxycyclophosphamide (4-HC) is an active derivative of CTX *in vivo*, the 4-HC was used to treat the breast cancer cells with different genotype of *GSTP1 105Val* allele *in vitro*. The 3-(4,5-dimethylthiazol-2-yl)-2,5-diphenyltetrazolium bromide (MTT) cell proliferation assays showed that the T47D cells with *Val*/*Val* genotype were more sensitivity to 4-HC-induced proliferation inhibition than the MCF-7 cells with *Ile/Val* genotype and MDA-MB-435 cells with *Ile*/*Ile* genotype (T47D *vs.* MDA-MB-435, *P* = 0.012; Figure 2C). Furthermore, the *GSTP1* non-expressing MCF-7 cells were transiently transfected with the *GSTP1 105Ile/Ile* and *GSTP1 105 Val/Val* GFP fusion plasmids, and than they were treated by 4-HC. The MTT cell proliferation assays confirmed that the *GSTP1 105Val*/*Val* genotype enhanced the sensitivity to 4-HC-induced proliferation inhibition compared to the *GSTP1 105Ile*/*Ile* genotype in MCF-7 transfected cells (*P* = 0.027; Figure 2D). The results indicate that *GSTP1 Ile105Val* affects breast cancer cell response to CTX *in vivo*.

## Discussion

The genotypic distributions of *GSTP1 Ile105Val* were evaluated based on a large cohort of women with breast cancer and healthy populations in North China. In the healthy control group, the genotypic distributions were 66.3% of *Ile*/*Ile*, 29.3% of *Ile*/*Val* and 4.4% of *Val*/*Val*. The frequency of *Val* (Ile/*Val* and *Val*/*Val*) alleles (33.7%) is similar to other reports of a Chinese population in Shanghai in Southeast China (33.2%) [15] and Taiwan (33%) [22], but lower than that reported in Indian (54.0%) [19], Slovakian (51.8%) [23], European-American (58%) and African-American populations (65%) [22], and a little higher than that for Englishmen (28%) [11] and Italian (30%) [24] These data indicate that the genotypic distributions of *GSTP1 Ile105Val* in Chinese populations differ from those in Western and certain other Asian populations. It is known that the allelic frequencies of metabolic genes are not equally distributed throughout human populations, and the frequencies might follow diverse ethnic and/or geographic-specific patterns [23].

Genetic polymorphisms in genes coding metabolic enzymes have been thought to be related to breast cancer susceptibility [25]. In this study, we observed that the *GSTP1 105Val* (Ile/*Val* and *Val*/*Val*) allele carriers had a higher risk of breast cancer than those with the homozygous Ile/Ile(OR = 1.38). These results are consistent with results from studies of Americans [17], Indians [18,19], and Chinese in Shanghai in Southeast China [16]. However, in several studies on African-Americans [20], white women in North Carolina [26] and Caucasians [21], no significant differences were found between the *GSTP1 Ile105Val* polymorphism and breast cancer risk. Studies of the Finnish [27] and Koreans [14] showed that the GSTP1 *105Val* allele was associated with a lower risk of breast cancer. A recent meta-analysis showed that *GSTP1 105Val* was associated with an increased breast cancer risk in Chinese populations but not in non-Chinese populations [28], which is consistent with our results. The GSTP1 enzyme plays an important role in the metabolism and inactivation of various toxic compounds [29]. The *Ile105Val* polymorphism is a transition from an A to a G in nucleotide position 313 (A313G), leading to an Ile105Val amino acid change located near the substrate binding site of the enzyme. The altered protein expression may lead to subsequent accumulation of carcinogens in the body, resulting in the development of a malignant phenotype. The environmental pollution in developing countries such as China and India caused by increasing population levels and changes in modern lifestyle may contribute to the increased breast cancer risk in those with the *GSTP1 105Val* allele.

In addition, we found that breast cancer patients with the *GSTP1 105Val* allele were more likely to bear a tumor with histological grade III, lymph node metastases, as well as ER negative than those carrying the *Ile*/*Ile* allele. Our evidence indicates that the *GSTP1* with the *105Val* variant lost or reduced enzyme activity compared with *Ile*/*Ile* genotype leading to the accumulation of toxic substances in the body. The toxic damage to genomic DNA in somatic cells not only induces carcinogenesis [30] but also causes tumors with more aggressive characteristics such as poor differentiation, hormone-independent growth and metastatic potential.

The *GSTP1 105Val* genotype is an unfavorable factor for healthy females, however, it is a favorable factor for the cytotoxic efficacy of chemotherapy for breast cancer patients. Thus, the patients with the *105Val* genotype may have better prognosis than those homozygous for *Ile*/*Ile*. The report in Shanghai revealed that breast cancer patients with the *GSTP1 105Val* allele had a 60% reduction in mortality risk after chemotherapy (HR = 0.4, 95% CI: 0.2-0.8) [30]. Our results further demonstrated that the *GSTP1 105Val* genotype provided a good prognosis for breast cancer patients in a Chinese population after receiving CTX-based chemotherapy (HR = 0.77, 95% CI: 0.45-0.91). However, this genotype was not associated with prognosis in breast cancer patients receiving CTX-containing chemotherapy in North American [31]. The GSTP1 enzyme exhibits specific and high activity in the conjugation of CTX and its toxic metabolites [32]. The SNP of *GSTP1 Ile105Val* substitutions in the coding sequence results amino acid changes within the GSTP1 substrate-binding site [10,33]. Evidence has demonstrated that the *GSTP1 105Val* variant is associated with a lower thermal stability and altered catalytic activity to a variety of substrates compared with *GSTP1 10*5*Ile* [30] and presents a reduced ability to detoxify chemotherapeutic agents, which results in lower clearance and better efficacy. We further investigated the effect of the *GSTP1 Ile105Val* genotype on breast cancer drug resistance through *in vitro* cellular experiments. The results confirmed that the breast cancer cells with the *GSTP1 105Val*/*Val* genotype exhibited increased sensitivity to 4-HC, which is an active derivative of CTX *in vivo*, than the cells with the *Ile/Ile* genotype. The results provide further evidence that the *GSTP1 Ile105Val* genotype affects the therapeutic response and survival of breast cancer patients treated with CTX.

In summary, our results demonstrated that among women in North China, the *GSTP1 105Val* allele carries a higher breast cancer risk and a risk of more aggressive tumors. However, patients with this allele have a trend toward improved survival after treatment with CTX-based chemotherapy. Therefore, the *GSTP1 Ile105Val* genotype could serve as a molecular test to screen for a high risk of breast cancer, to evaluate breast cancer aggressiveness and to predict the efficacy of CTX-based chemotherapy in Chinese populations. Due to the limited number of cases with rare genotype *Val*/*Val* of *GSTP1 lle105Val* in this study, the preventive, diagnostic and therapeutic values of the genetyping for women and breast cancer patients should be further evaluated and confirmed by large multicenter studies.

## Materials and Methods

### Patients

A total of 920 breast cancer patients (aged 24-65 years, mean age 54.3 years) and 783 healthy women (aged 21-69 years, mean age 53.2 years) were recruited for this study. A *t*-test indicated that the mean age of these two population groups was equal (*P* = 0.120). All patients and control individuals were genetically unrelated women from North China. A total of 796 patients underwent unilateral mastectomy and dissection of axillary lymph nodes in Tianjin Medical University Cancer Institute and Hospital (TMUCIH; Tianjin, China) from January 2005 to January 2007, and diagnoses were confirmed based on pathological examinations. The remaining 124 patients were post-surgical re-examination at TMUCIH. All cases were diagnosed as invasive carcinoma. Detailed clinicopathological information including patient age, clinical stage, tumor size, histological grade, lymph node involvement, as well as estrogen receptor (ER), progesterone receptor (PR), and human epidermal growth factor receptor 2 (HER2) statuses are presented in Table 5. ER, PR and HER2 statuses in breast cancer tissues were determined through immunohistochemical staining. All patients received CTX-based chemotherapy for at least 4 cycles. CTX was administered through an intravenous injection line. The doses were within the standard range of 500–600 mg/m^2^. The imaging examination (ultrasound, X-ray, MRI, ECT and CT) and/or pathological diagnosis were performed to monitor for DFS status in the follow-up. DFS was defined as the time interval between primary surgery and any relapse (local-regional, contra-lateral and/or distant), or terminal time of follow-up without any relapse events. 879 of 920 cases were followed-up with over five years. This study was approved by the Institutional Review Board of TMUCIH, and written consent was obtained from all participants.

**Table 5:** Clinicopathological characteristics of breast cancer patients.

**Characteristics**		**Cases (%)**
**Age (year)**	< 55	587 (63.8)
	> = 55	333 (36.2)
**Clinical stage**	I+II	717 (77.9)
	III+IV	166 (18.0)
	Unknown	37 (4.1)
**Lymph node status**	Negative	647 (70.3)
	Positive	219 (23.8)
	Unknown	54 (5.9)
**Histological grade**	I+II	605 (65.7)
	III	217 (23.6)
	Unknown	98 (10.7)
**ER status**	Positive	566 (61.5)
	Negative	248 (27.0)
	Unknown	106 (11.5)
**PR status**	Positive	583 (63.4)
	Negative	231 (23.1)
	Unknown	106 (11.5)
**HER2 status**	Negative	626 (68.1)
	Positive	188 (20.4)
	Unknown	106 (11.5)
**5-years DFS**	Negative	619 (67.3)
	Positive	260 (28.3)
	Unknown	41 (4.4)

Note: “Unknown” represents the number (%) of cases for which the corresponding information was not available.

### Cell culture

The breast cancer cell lines MCF-7, T-47D, MDA-MB-468, MDA-MB-231 and MDA-MB-435 were obtained from the American Type Culture Collection (ATCC). The cells were cultured in RPMI 1640 (MCF-7 and MDA-MB-231) or DMEM/F12 (T-47D, MDA-MB-468 and MDA-MB-435) medium supplemented with 10% fetal bovine serum, 100 units/mL penicillin and 100 µg/mL streptomycin (Invitrogen, Carlsbad, CA, USA) and incubated in a humidified atmosphere with 5% CO_2_ at 37°C.

### Specimens and genomic DNA extraction

A volume of 2 mL of peripheral blood was collected from each individual and treated with EDTA-K2 anticoagulant. Nucleated cells were then separated by hypotonic lysis of red blood cells as described previously [34]. Genomic DNA from nucleated cells and cultured cells was extracted according to standard methods using proteinase K followed by phenol/chloroform/isopropanol treatment or using QIAamp DNA Blood Mini Kit (Qiagen, Valencia, CA, USA). DNA concentrations were determined with a UV spectrophotometer. DNA integrity and purity were assayed through 1.5% agarose gel electrophoresis. TE buffer (10 mM Tris-HCl and 1.0 mM EDTA, pH 8.0) was used for resuspending DNA, with the final concentration adjusted to 200-500 ng/µL. The DNA solutions were frozen and stored at −80°C.

### Genotyping by the TaqMan allelic discrimination assay

The primers and TaqMan probes for the genotypic analysis of *GSTP1 Ile105Val* were designed and optimized using Oligo 6.0 software (Molecular Biology Insights, West Cascade, USA), and synthesized by Sangon Biological Engineering Technology & Services Co, Ltd. (Shanghai, China). The PCR-TaqMan allelic discrimination assays were performed using the Platinum Quantitative PCR SuperMix-UDG System (Invitrogen) according to the manufacturer’s instructions with the ABI 7500 TaqMan system (Applied Biosystems, Carlsbad, CA, USA).

### DNA sequencing

To validate the data generated by PCR-TaqMan assay, 10% of the samples were randomly sequenced. The sequencing reactions were performed according to the conventional dideoxy chain-termination method using an ABI PRISMTM 3130 Genetic Analyzer (Applied Biosystem).

### Construction and transfection of *GSTP1 105 Ile* and *Val* expression plasmids

The GSTP1 *Ile105Val* genotypes of the breast cancer cells MCF-7, T-47D, MDA-MB-231 and MDA-MB-435 were detected using the TaqMan allelic discrimination assay and were confirmed through DNA sequencing. PCR was performed to amplify *GSTP1 105Ile* (A/A) and *Val* (G/G) full-length cDNAs from cell lines with the corresponding genotype. The primers were 5’-CCAAGCTTACCATGCCGCCCTACACC-3’ (forward) and 5’-CCGGATCCTGTTTCCCGTTGCCAT-3’ (reverse), with BamHI and HindIII restriction endonuclease recognition sites (underlined) on the 5’ ends. The PCR reactions were performed in a volume of 50 µL at 95°C for 2 min and 35 cycles of 95°C for 30s, 58°C for 1 min, and 72°C for 1 min, followed by 72°C for 10 min. The PCR products were subcloned into the pCR2.1 plasmids (Invitrogen) and expanded in DH5 alpha *E. coli*. Full-length cDNAs with *GSTP1 105Ile* and *Val* genotypes were digested from pCR2.1 using restriction endonucleases and subcloned into the NH2-terminus of green fluorescent protein (GFP) of the mammalian expression plasmid pEGFP-N1 (Clontech, Palo Alto, CA, USA). The *GSTP1s* sequences in the recombinant plasmids were confirmed through DNA sequencing, and GSTP1s expression levels were detected using reverse transcription-quantitative polymerase chain reaction (RT-QPCR) and western blot assays. For transient transfection, 2×10^5^ MCF7 cells per well in 6-well plates were cultured without antibiotics overnight and then transfected with recombinant plasmids using Lipofectamine 2000 reagent (Invitrogen) according to the manufacturer’s instructions.

### RT-QPCR

Total RNA from cultured cells was extracted with TRIZOL reagent, reverse transcription (RT) was performed using the SuperScript First-Strand cDNAs Synthesis kit, and real-time quantitative PCR (QPCR) was performed using Platinum SYBR Green qPCR SuperMix-UDG. All reagents were Invitrogen products, and the reactions were performed according to the manufacturer’s instructions. The primers for *GSTP1* cDNA amplification were 5’-AGGACCTCCGCTGCAAATACATCT-3’ and 5’-TCTCCCACAATGAAGGTCTTG-3’. The primers for the housekeeping gene glyceraldehyde 3-phosphate dehydrogenase (*GAPDH*) were as previously described [35]. QPCR was performed with the parameters of 50°C for 2 min, pre-denaturation at 95°C for 3 min, and 45 cycles at 95°C for 30 sec and 62°C for 1 min. Target gene expression quantification in samples was accomplished by measuring the fractional cycle number at which the amount of expression reached a fixed threshold (C_T_). Triplicate C_T_ values were averaged, and *GAPDH* C_T_ was subtracted from *GSTP1* C_T_ to obtain ΔC_T_. The relative amount of *GSTP1* mRNA was calculated as 2^-ΔCT^.

### Western blot

Cultured cells were solubilized with protein lysis buffer. The proteins were separated by size using SDS-PAGE and transferred to polyvinyldifluoride membranes (Pierce, Rockford, IL, USA). The membranes were blocked with 5% milk in TBST (10 mM Tris, 150 mM NaCl, 0.05% Tween 20, pH 8.3) for 60 min at room temperature and incubated with a 1:1000 dilution of rabbit polyclonal anti-GSTP1 antibody (Sigma, St. Louis, MO, USA) in TBST-milk overnight at 4 °C. Non-bound primary antibody was removed by washing in TBST, and bound antibody was detected using HRP-conjugated goat anti-rabbit IgG. The immunoreactive protein bands were visualized by enhanced chemiluminescence (ECL) reagents (GE Healthcare).

### MTT assay

To assess the effect of the *GSTP1 Ile105Val* genotype on anti-cancer drug resistance, MDA-MB-435 (Ile/Ile), MCF-7 (Ile/Val) and T47D (Val/Val) cells lines were assessed using the MTT assay. T47D cells (Val/Val), MDA-MB-435 cells (Ile/Ile) and MCF-7 cells (Ile/Val) were treated by 4-HC and analyzed the cell proliferation using MTT assay. MCF-7 cells, which do not express GSTP1, were transfected with pEGFP-GSTP1 105Ile (Ile/Ile), pEGFP-GSTP1 105Val (Val/Val), and the vector control pEGFP-N1, and then also were treated by 4-HC and analyzed using MTT assay. All the cells were plated at 1×10^4^ cells per well in 96-well plates and incubated with 15 µg/mL 4-HC for 24, 48 and 72 h. Then, 10 µL of MTT (5 g/L in PBS) was added to each well and incubated at 37 °C for 4 h, and the medium was replaced by 100 µL DMSO to dissolve the formazan. Absorbance was measured at 570 nm using a spectrophotometer. Cell viability was calculated as the value relative to control cultures. The cells were tested in three independent assays with each containing triplicates

### Statistical analyses

The Chi-square (χ^2^) test or Fisher’s exact test was used to compare the SNP genotypic distributions between the breast cancer group and the healthy controls, among cancer patients with various clinicopathological parameters, and for analysis of the Hardy-Weinberg equilibrium. Polytomous logistic regression was used to estimate the odds ratios (OR) and 95 percent confidence intervals (95% CI) as measures of association between the genotypes and breast cancer risk subtypes or to compare case subtypes to all controls. Survival analyses were performed according to the Kaplan and Meier methods and assessed using the log-rank test. All prognostic variables in the multivariate survival analysis were performed using a backward stepwise Cox proportional hazards regression model, and hazard ratio (HR) and relative risk (RR) were calculated from the Cox model. Statistical analyses were performed with the Statistical Package for the Social Sciences (SPSS, version 19.0). *P*-values less than 0.05 were considered statistically significant. All calculated data of experiments *in vitro* are expressed as mean ± standard deviation (SD).
